# Comparison of Clinical Features of Bipolar Disorder Patients with and without Psychiatric Comorbidity

**DOI:** 10.5152/eurasianjmed.2021.20270

**Published:** 2021-10

**Authors:** Tonguc Demir Berkol, Hasan Mervan Aytac

**Affiliations:** 1 Department of Psychiatry, Bakırköy Research and Training Hospital for Psychiatry, Neurology and Neurosurgery, İstanbul, Turkey; 2 Department of Psychiatry, Başakşehir Çam and Sakura City Hospital, İstanbul, Turkey

**Keywords:** Bipolar disorder, comorbidity, axis-I, anxiety disorders, prognosis

## Abstract

**Objective:**

Bipolar disorder (BPD) is a psychiatric condition that often manifests together with Axis-I comorbidity. Comorbidity of psychiatric disorders influences the recognition, prognosis, and treatment of the disorder, posing difficulties for the patient and physician. This study aims at identifying Axis-I comorbidities and their characteristics in patients with BPD.

**Materials and Methods:**

This retrospective study included 255 patients diagnosed with BPD according to the DSM-IV. Comorbidities were determined using the SCID-I, a semistructured sociodemographic data form developed by the authors, and a mood chart assessing clinical aspects. The patients were divided into 2 groups, those with and without comorbidity, and compared.

**Results:**

Out of these 255 patients, 35% was found to have a current and 84.3% a lifelong comorbid psychiatric disorder. About 33.1% of these patients had 1, 11.3% had 2, and 8.8% had more than 2 comorbid disorders. At least one comorbid anxiety disorder was found in 38.7% of the patients. Obsessive and compulsive disorder (OCD) being the most common comorbid psychiatric disorder, followed by social anxiety disorder (SAD), posttraumatic stress disorder (PTSD), and generalized anxiety disorder (GAD). Comparing the clinical parameters between the 2 groups due to the presence of psychiatric comorbidity in the BD patients, there were statistically significant differences in terms of the number of depressive episodes (*P* = .041) and mania/hypomania (*P* = .048), and the need for antipsychotic monotherapy (*P* = .007) and antidepressants (*P* = .001) for prophylaxis between the 2 groups.

**Conclusion:**

Axis-I psychiatric disorders and particularly OCD and anxiety disorders accompanying BPD prevail at a high rate. The presence of comorbid psychiatric disorder in BPD may negatively affect the clinical prognosis of the condition; therefore, this area requires more study and careful investigation.

## Introduction

Bipolar disorder (BPD) is a psychiatric condition that involves episodic and recurrent episodes of mania, hypomania, depression, or mixed episodes with possible remissions between episodes. It affects functioning and has high rates of mortality and morbidity.^1^ BPD’s lifelong prevalence has been reported to be 1%, ranging between 0 and 2.4 for BPD type-I and between 0.3 and 4.8 for BPD type-II. It is reported to start more commonly at ages 15–19 and rarely before 12 or after 60 years of age.^2^ Its prevalence has been found at similar rates in females and males.^3-^^5^

The risk of psychiatric comorbidity is known to be high in individuals diagnosed with BPD. Many studies have shown that the risk of having psychiatric comorbidity is higher than 50% in patients with BPD.^6-^^8^ The most common psychiatric comorbidity is anxiety disorders,^9^ but it is still unknown which is more common in BPD patients. Various studies have reported that the most common psychiatric disorders seen among patients with BPD are obsessive-compulsive disorder (OCD) with 39%^7^ and 35%,^8^ social anxiety disorder (SAD) with 13% and 39%,^10^ panic disorder (PD) with 20%,^6^ and generalized anxiety disorder (GAD) with 20%.^11^ One of the critical difficulties clinicians face is that most BPD patients simultaneously meet more than one anxiety disorder criterion.

The presence of a comorbid anxiety disorder in patients with BPD is argued to influence some sociodemographic and clinical outcomes. In a study, BPD patients with anxiety disorder comorbidity were observed to be younger; mainly, male patients had the disorder at an earlier age, and their education statuses were inferior.^8^ Additionally, BPD patients with a comorbid anxiety disorder exhibited an inadequate response to treatment, a high rate of suicide attempts, more hospitalization, longer-lasting episodes, more psychotic characteristics and mixed episodes, and higher rates of substance addiction.^8,12,13^ The prevalence of any anxiety disorder, OCD, SAD, and substance abuse were found significantly higher in patients with BPD who had a mood disorder in their family than those who did not have such a family history.^14^

About 25–50% of the BPD patients attempt to commit suicide, and 15% lose their lives due to such attempts.^15^ One of our previous studies revealed that the female gender, a history of mood disorder in the family, and eating disorder were more common in BPD patients who had at least one suicide attempt.^16^ Our study hypothesized that the most common comorbid psychiatric disorder of BPD would be anxiety disorders, and the prognosis of BPD patients with comorbid disease would be worse. The present study aims to evaluate comorbid Axis-I disorders in patients with BPD and reveal the clinical outcomes of psychiatric comorbidity in BPD’s progress and outcomes.

## Materials and Methods

After receiving their consent, 255 BPD patients (244 BD-I and 11 BD-II) were followed up from Haseki Training and Research Hospital Psychiatry Outpatient Clinic between March 2016 and May 2017 was included in the study. SCID-I/CV (Structured Clinical Interview for DSM-IV/Clinical version)^17,18^ was applied to all participating patients. The patients were divided into 2 groups due to lifelong comorbid diagnoses and named a BPD with and without comorbidity. The patients with BPD were admitted to outpatient clinic and agreed to participate in our study with written informed consent. The ethics committee of our study was taken under the current Declaration of Helsinki from Üsküdar University Non-Interventional Research Ethics Board (Number: B.08.6.YÖK.2.ÜS.0.05.0.06/2016/57-Date: 25.04.2016).

The study was designed retrospectively. However, the treatment records were obtained from a BPD follow-up program, which also covered their treatment schedule. Semistructured interview charts that assessed patients’ sociodemographic and clinical features (age of onset of disease, family history, presence of psychotic features, number, and type of episodes, type, duration, results of the treatment, etc.) were filled with information from the patients, their families, and previous psychiatric admission records. Mood charts including graphical records for the disorders’ and treatments’ outcome since the onset of the BPD (The age at onset of the disease was taken as the age at which the DSM-IV mood episode met the criteria for the first time.) were filled out as well. Both charts were updated in every follow-up visit. During the follow-ups, patients and relatives were interviewed once a month in the first 6 months, once every 2 months in the following 6 months, and for remitted patients, once every 3 months in the rest of the maintenance period. In cases of recurrence, follow-up visits were made more frequently according to the needs of the patient and prescribed treatment. Patients’ mood charts and all medical records were reviewed for the present study, and missing sections were completed in follow-up visits when necessary.^19^

### Inclusion and Exclusion Criteria

Subjects of 18–65 years of age, of either gender, were literate, agreed on the participation in the study, diagnosed with a BD according to the SCID-I interview, had no other systemic/neurological disease that may affect cognitive functions included in the study. However, the patient with mental retardation, a psychiatric disorder due to general medical condition, receiving medical treatment, and alcohol or substance abuse and again the patient whose language and education level not sufficient to perform psychiatric interview were excluded from the study.

### Statistical Analysis

In this study, the statistical analyses were conducted using IBM SPSS Statistics for Windows, Version 11 (SPSS Inc.; Chicago, IL, USA). Chi-square test was used to establish the relationship between psychiatric disorder comorbidity and clinical characteristics. Continuous variables (sociodemographic and clinical parameters) were analyzed using 2 independent samples Student t-test. All *P* values were 2-sided, and the statistical significance was set to *P* < .05.

## Results

### Comparison of Socio-Demographic Characteristics of BPD Patients due to Comorbid Psychiatric Disorder

Of the patients who participated in the study, 65.9% were female and 34.1% male. The mean age was 40.7 ± 12.6 years. The proportion of BPD patients with a comorbid disorder who went to a university was 52.9% and without a comorbid disorder, 47.1%. No significant difference was found between the sociodemographic characteristics of the 2 groups (Tables 1 and 2).

### Comparison of Clinical Parameters of BPD Patients due to Comorbid Psychiatric Disorder

There was no difference between the groups in terms of onset of BPD (*P* = .772), number of episodes (*P* = .998), first and dominant episode types (*P* = .236), history of BPD in first-degree relatives (*P* = .524), severity of episodes (*P* = .776), hospitalization (*P* = .543), response to lithium and anticonvulsant therapies (respectively *P* = .906, *P* = .964), and psychotic (*P* = .684), chronic (*P* = .378), and suicidal tendencies (*P* = .141) (Tables 1 and 2).

The BPD patients with comorbidity were observed to have significantly higher rates of depressive episodes (*P* = .041) and mania/hypomania (*P* = .048), and they used significantly more antipsychotic monotherapy (*P* = .007) and antidepressants (*P* = .001) for prophylaxis (Tables 1 and 2).

### The Percentages of Current or Lifelong Comorbid Psychiatric Disorder in BPD Patients

A current comorbid psychiatric disorder was present in 34.9% of the BPD patients (n = 89), whereas 84.3% had a lifelong comorbid psychiatric disorder (n = 215). Of these patients, 33.1% had 1 comorbid disorder, 11.3% had 2, and 8.8% had 3. At least 1 comorbid anxiety disorder was present in 38.7% of the patients. The most frequently seen lifelong psychiatric comorbidity in patients with BPD was OCD (54/255, 21.2%) followed by SAD (45/255, 17.5%), posttraumatic stress disorder (PTSD) (38/255, 15.0%), and GAD (22/255, 8.7%) (Table 3).

## Discussion

In our study, the prevalence of lifelong comorbid anxiety disorders in BPD patients is higher than the other psychiatric disorders comorbidity, compatible with the literature. Recent epidemiological and clinical studies have shown that BPD is accompanied by Axis-I psychotic disorders and particularly anxiety disorders at a high rate. The prevalence of a comorbid psychiatric disorder with BPD is around 60.8% and 65%.^6^ However, information on how these comorbidities differed concerning patients’ clinical, sociodemographic, and treatment-related characteristics is still insufficient.^20^ Many studies have shown that more than half of BPD patients have at least one anxiety disorder.^7,8^ In a study, the BPDs were present in 8.7% of the patients with panic disorder, followed by agoraphobia (19%), SAD (16.1%), GAD (25.3%), and OCD (13.8%).^21^ A meta-analysis that included 52 studies reported that panic disorder was the most common lifelong anxiety disorder accompanying BPD with 16.8%.^10^ The most common comorbid psychiatric disorder was OCD in our study and also in 2 other studies made in our country.^7,22^ The BPD patients with comorbid OCD were characterized with more suicide attempts, less response to OCD treatment, and later onset of OCD symptoms than those diagnosed with OCD alone, and the symptoms were periodic and more frequent in males.^23,24^ There were individuals diagnosed with BPD who also had comorbidity. The BPD patients with comorbid OCD were found to have more sexual, religious, aggressive, and impulsive obsessions and more controlling, collection, and repetition compulsions.^24^ Studies based on follow-up of the children of patients diagnosed with BPD have reported that anxiety disorders may be BPD’s precursor before the disease is diagnosed.^25,26^ We found fewer comorbidity rates in our study than in the literature, although alcohol and substance use disorders are common in BPD patients.^6^ Such low rates may be due to some cultural factors and because a large number of female patients who are known to have lower rates of alcohol and substance use disorder in our country participated in our study, and patients usually hesitate to disclose their alcohol and substance use.

The presence of comorbid anxiety disorder is thought to affect BPD’s course and prognosis negatively.^12^ The presence of apparent anxiety or an anxiety disorder in patients with BPD has been observed to increase an earlier age of onset, suicide attempts, psychotic characteristics, hospitalization, depressive episodes, and the rate at which below-threshold mood characteristics between episodes become chronic.^7,8,13,20^ Our results also support these reports. For this reason, our study is not consistent with the fewer studies arguing that the presence of anxiety comorbidity may not have a direct negative effect on the clinical prognosis in patients with BPD.^27^

Our study shows that a higher depressive episode rate in BPD patients with comorbidity is compatible with the literature. A large majority of patients with an anxiety disorder are known to have also depressive disorders. The presence of a comorbid anxiety disorder in BPD patients who experience depressive and mixed episodes more frequently^8,28^ supports this result. These results support the use of antidepressants at a high rate in the BPD-comorbid group in our study.

Our results also underline the fact that the presence of a comorbid psychiatric disorder negatively affects the response to treatment as well as the clinical prognosis of BPD. Very few studies have investigated the effect of a comorbid psychiatric disorder on pharmacological treatment efficacy in BPDs. Studies report that a comorbid SAD may not be associated with response to treatment in BPD patients.^29^ Some studies showed that response to lithium was low in BPD patients with a high anxiety grade,^12,13^ and another study showed that there was no difference in response to lithium therapy in BPD patients with a comorbid anxiety disorder, but their response to an anticonvulsant therapy was lower.^27^ Mixed periods accompanied by high anxiety levels are reported to respond better to valproate than to lithium.^30^

The low rate of suicide attempts in this study may be linked to the inclusion of patients who were still on treatment at outpatient clinics in the study. Patients at a high risk of suicide may have been hospitalized.^31^ One of the most important limitations of our article is that it was made retrospectively. Besides, the fact that the sample group was taken only from Istanbul can be stated as another limitation. The number of patients should be taken into consideration when comparing data to those in the literature. Such issues should be considered as the limitations of a study.

In conclusion, we have seen that the results of the present study confirm our hypothesis. Psychiatric comorbidity, and particularly an anxiety disorder, is frequently encountered in BPD. In our study, at least one comorbid anxiety disorder was present in 38.7% of the patients. The most frequent comorbid psychiatric disorder was OCD. The presence of a comorbid disorder increases the number of depressive episodes, mania/hypomania, and the use of antidepressants and antipsychotics, affecting the clinical prognosis of BPD or response to treatment.

Possible effects of different diseases could not be evaluated because the effect of a comorbid disorder appeared as an entity involving all psychiatric disorders. A comorbid anxiety disorder accompanying to BPD may be affecting the clinical prognosis of the condition negatively. For this reason, there is a need for further studies to assess the effects of various disorders on the clinical prognosis of BPD and especially on the pharmacological treatment of it. Besides, with future studies about BPD’s neurobiology, we hope to begin to improve our look at the psychiatric comorbidity. Progressing the pathophysiological links between specific psychiatric disorders and BPD, including the use of clinical biomarkers to help refine the understanding of BPD, may help clarify the pathophysiology of BPD itself. These types of studies will eventually suggest new patterns for secondary prevention and long-term treatments.

**Main Points**

- The most frequent comorbid psychiatric disorder of BPD was OCD.
- Anxiety disorders accompanying BPD prevail at a high rate.
- The presence of a comorbid disorder increases the number of depressive episodes, mania/hypomania, and the use of antidepressants and antipsychotics.

## Figures and Tables

**Table 1.** Differential Qualitative Characteristics of Bipolar Patients with and without a Comorbid Disorder

	Bipolar Disorder: Group with Comorbidity (n = 135), n (%)	Bipolar Disorder: Group without Comorbidity (n = 120), n (%)	*P* ^*^
Bipolar I	127 (94.1)	117 (97.5)	.179
Bipolar II	8 (5.9)	3 (2.5)	
Dominant episode			
Depressive	34 (25.2)	21 (17.5)	.236
Manic	87 (64.4)	89 (74.2)	
Mixed	14 (10.4)	10 (8.3)	
Psychotic symptoms (+)	102 (75.6)	88 (73.3)	.684
Suicide attempts (+)	30 (22.2)	18 (15.0)	.141
Lithium monotherapy			
There is response	71 (52.6)	64 (53.3)	.906
No Response	64 (47.4)	56 (46.7)	
Anticonvulsant monotherapy			
There is response	84 (62.2)	75 (62.5)	.964
No response	51 (37.8)	45 (37.5)	
Antidepressant Use (+)	83 (61.5)	48 (40.0)	.001
Antipsychotic Use (+)	90 (66.6)	60 (50.0)	.007
Bipolar disorder in relatives (+)	35 (25.9)	27 (22.5)	.524
Hospitalization	86 (63.7)	72 (60.0)	.543
Mean episode severity			
Mild	55 (40.7)	51 (42.5)	.776
Severe	80 (59.3)	69 (57.5)	

* Pearson chi-square

**Table 2.** Sociodemographic and Clinical Characteristics of Bipolar Patients with and without a Comorbid Disorder

	Bipolar Disorder: Group with Comorbidity (n = 135), mean (SD)	Bipolar Disorder: Group without Comorbidity (n = 120), mean (SD)	*P* ^**^
Age (years)	39.9 (12.1)	41.6 (13.2)	.287
Age at onset (years)	23.6 (8.5)	23.9 (8.0)	.772
Mean length of cycle	12.8 (8.2)	13.8 (9.7)	.378
Total number of episodes	9.3 (6.7)	9.3 (7.8)	.998
Polarity rates of episodes			
Manic/hypomania	81 (67.5)	75 (55.6)	.048
Depressive	47 (34.8)	28 (23.3)	.041
Mixed	13 (9.6)	11 (9.2)	.899

** Independent samples *t*-test, SD: standard deviation

**Table 3.** Axis-I psychiatric comorbidity rates in bipolar disorder

	SCID-I current		SCID-I lifelong	
Diagnosis (Entire patients n= 255)	n	%	n	%
Alcohol addiction	6	2.50	11	4.38
Panic disorder	6	2.50	13	5.00
Social anxiety disorder	13	5.00	45	17.50
Specific phobia	10	3.75	13	5.00
Obsessive-compulsive disorder	29	11.25	54	21.25
Post-traumatic stress disorder	0	0.00	38	15.00
Generalized anxiety disorder	18	6.88	22	8.75
Anxiety disorder, not otherwise specified	6	2.50	6	2.50
Somatoform disorder	0	0.00	6	2.50
Eating disorder	2	0.63	6	2.50
**Total**	**89**	**34.9**	**215**	**84.3**

SCID-I: Structured Clinical Interview for DSM-IV axis-I
